# PLASMA PROTEOMIC SIGNATURE OF DEVELOPMENT AND PROGNOSIS OF PHYSICAL FRAILTY

**DOI:** 10.1093/geroni/igae098.2770

**Published:** 2024-12-31

**Authors:** Jianhong Xu, jingyun Liu, Jinhui Liao, Chenkai Wu

**Affiliations:** Duke Kunshan University, Kunshan, Jiangsu, China (People’s Republic); Duke Kunshan University, Kunshan, Jiangsu, China (People’s Republic); Duke Kunshan University, Kunshan, Jiangsu, China (People’s Republic); Duke Kunshan University, Kunshan, Jiangsu, China (People’s Republic)

## Abstract

The complete understanding of biological mechanisms behind frailty remains elusive. We aimed to investigate the proteomic signature associated with the development and prognosis of frailty. Data from 43,895 participants (aged 39-70 years) in the UK Biobank were analyzed, with 2,920 proteins assayed using Olink. Participants were split into development and validation cohorts based on geographic location. Multinomial logistic models identified proteins cross-sectionally associated with baseline prefrailty and frailty (Bonferroni-corrected p-value < 0.05). Cox models identified proteins associated with mortality among initially prefrail and frail participants. LASSO regression was used to construct protein-based prediction models for death among prefrail and frail participants, respectively. Protein importance was ranked using the light gradient boosting machine classifier. We identified 210 and 268 proteins cross-sectionally associated with prefrailty and frailty at baseline, respectively. Additionally, 109 and 80 proteins were associated with mortality among initially prefrail and frail individuals, respectively. LASSO regression model selected 60 and 21 proteins predicting survival in prefrail and frail participants, respectively. Protein-based prediction models demonstrated satisfactory discrimination, calibration, and reclassification in the development and validation cohorts. GDF15 (WFDC2) and PLAUR emerged as the top two proteins in prefrailty (frailty) development, demonstrating robust connections with overall survival in prefrail (frail) individuals. Differentially expressed proteins were enriched in pathways involving protein metabolism, cellular signaling in disease, apoptosis, and inflammation. Exploring novel proteins and pathways provides insights into the biological mechanisms and potential therapeutic targets associated with the onset and progression of frailty.

